# Barcoding Poplars (*Populus* L.) from Western China

**DOI:** 10.1371/journal.pone.0071710

**Published:** 2013-08-19

**Authors:** Jianju Feng, Dechun Jiang, Huiying Shang, Miao Dong, Gaini Wang, Xinyu He, Changming Zhao, Kangshan Mao

**Affiliations:** 1 School of Earth and Environmental Sciences & State Key Laboratory of Grassland Agro-Ecosystem, School of Life Sciences, Lanzhou University, Lanzhou, Gansu, People’s Republic of China; 2 College of Plant Sciences, Xinjiang Production & Construction Corps Key Laboratory of Protection and Utilization of Biological Resources in Tarim Basin, Tarimu University, Alar, Xinjiang, People’s Republic of China; George Washington University, United States of America

## Abstract

**Background:**

*Populus* is an ecologically and economically important genus of trees, but distinguishing between wild species is relatively difficult due to extensive interspecific hybridization and introgression, and the high level of intraspecific morphological variation. The DNA barcoding approach is a potential solution to this problem.

**Methodology/Principal Findings:**

Here, we tested the discrimination power of five chloroplast barcodes and one nuclear barcode (ITS) among 95 trees that represent 21 *Populus* species from western China. Among all single barcode candidates, the discrimination power is highest for the nuclear ITS, progressively lower for chloroplast barcodes *mat*K (M), *trn*G-*psb*K (G) and *psb*K-*psb*I (P), and *trn*H-*psb*A (H) and *rbc*L (R); the discrimination efficiency of the nuclear ITS (I) is also higher than any two-, three-, or even the five-locus combination of chloroplast barcodes. Among the five combinations of a single chloroplast barcode plus the nuclear ITS, H+I and P+I differentiated the highest and lowest portion of species, respectively. The highest discrimination rate for the barcodes or barcode combinations examined here is 55.0% (H+I), and usually discrimination failures occurred among species from sympatric or parapatric areas.

**Conclusions/Significance:**

In this case study, we showed that when discriminating *Populus* species from western China, the nuclear ITS region represents a more promising barcode than any maternally inherited chloroplast region or combination of chloroplast regions. Meanwhile, combining the ITS region with chloroplast regions may improve the barcoding success rate and assist in detecting recent interspecific hybridizations. Failure to discriminate among several groups of *Populus* species from sympatric or parapatric areas may have been the result of incomplete lineage sorting, frequent interspecific hybridizations and introgressions. We agree with a previous proposal for constructing a tiered barcoding system in plants, especially for taxonomic groups that have complex evolutionary histories (e.g. *Populus*).

## Introduction

The species within the genus *Populus* (collectively known as poplars) are one of the world’s most important groups of forest trees: they are widespread and play a significant role in the ecosystem of temperate and boreal forests across the northern hemisphere [1]–[2]. In addition, because of their fast growth rates, profuse vegetative propagation, adaptability to various ecological conditions and the numerous uses for their wood (e.g. timber, paper pulp and bio-energy resources), species of this genus are widely cultivated and exploited [1], [3]–[4]. Meanwhile, poplars have become a model organism for the study of tree biology [5]–[7] and publication of the full genome of western black cottonwood (*P. trichocarpa*) has attracted even more research into this group [6]–[8]. From an economical view of point, the wild *Populus* species undoubtedly provide the most critical breeding resource in the future [1], [3]–[4]. However, identification of the wild *Populus* species is still difficult because of their extensive interspecific hybridization and high levels of intraspecific morphological variation [2], [9]–[11]. The recently developed DNA barcoding approach is one way to address this problem.

DNA barcoding aims to achieve accurate species identification by sequencing a standard region of DNA [12]–[16]. The mitochondrial *CO1* (cytochrome *c* oxidase subunit 1) gene has been found to be highly efficient for discriminating animal species, including amphibians (e.g. [17]), birds (e.g. [18]) and fishes (e.g. [19]). However, in plants, mitochondrial regions are unsuitable for this approach because of their low mutation rates [20]–[21] and the search for suitable candidates has instead focused on chloroplast and nuclear gene fragments [14]–[16], [22]–[24]. Based on assessments of recoverability, sequence quality, and levels of species discrimination, the CBOL Plant Working Group [15] recommend the two chloroplast locus combination of *mat*K+*rbc*L as the core barcode for land plants, and *trn*H-*psb*A and the nuclear ribosomal internal transcribed spacer (ITS) as being complementary. A recent survey involving a larger collection of samples argued that the ITS (or ITS2) region should also be included in the core barcode for seed plants [16]. In addition to choosing barcoding markers, sampling of many individuals within each species is also essential for establishing a reference database for universal application [16].

In *Populus*, the identification of different species and clones is in continuous development. Previous studies demonstrated that isozyme markers are useful for the differentiation of the sections *Aigeiros*, *Tacamahaca* and *Populus* (e.g. [25]–[26]), and nuclear simple sequence repeats (SSRs) as well as amplified fragment-length polymorphism (AFLP) markers have a high potential to discriminate between clones [27]–[31]. Meanwhile, some nuclear genes, such as *PPO*, *LEAFY*, *GA20* oxydase or *CAD*-like, are capable of differentiating species and hybrids [10], [32]. Recently, Schroeder et al. [11] tested 40 chloroplast barcoding markers in seven widely cultivated poplar species and found that the combination of two intergenic spacers (*trn*G-*psb*K, *psb*K-*psb*I) and the coding region *rpo*C had the highest discrimination power. Wang et al. [33] surveyed the population genetics of two poplar sister species from the arid area of western China and suggested that the major ITS genotype clearly differentiated this species pair.

The genus *Populus* is dioecious, and since the pollen and seeds (usually small and numerous) are dispersed by wind, interspecific hybridization and introgression occur intensively and extensively among sympatric (or parapatric) species. This can occur between naturally co-existing species [33]–[35] or between exotic (cultivated) and native species [36]–[38]. Therefore, it would be most practical to test barcode markers in a group of species that are native to a particular area, although it is necessary to perform a preliminary screening of barcode markers among a group of widely cultivated (but well diverged) species that are naturally distributed across different areas. In this study we used several widely accepted plant barcoding markers (chloroplast: *mat*K, *rbc*L and *trn*H-*psb*A; nuclear: ITS) as well as two efficient chloroplast intergenic spacers (*trn*G-*psb*K and *psb*K-*psb*I) to delimitate 95 samples, representing 21 *Populus* species, comprising more than eighty percent of the native popular species that occur in western China. We aimed to (1) test the discrimination power of these barcode markers, alone or in combination; (2) establish a reference database to facilitate future identification of *Populus* species from this area; and (3) propose a way forward for the development of a highly efficient species identification approach in this genus.

## Materials and Methods

### Ethics Statement

All leave samples employed in this study were collected from tree species that are not endangered, and these trees grow in public area where no permission for collection of leaves is needed in China.

### Sample Collection

Leaves of 95 trees representing 21 poplar species were collected from western China (Table S1). Fresh leaves were dried and stored in silica gel, and the latitude, longitude and altitude of each collection site were recorded using an eTrex GIS unit (Garmin, Taiwan). Among these species, *P. × canescens* (Aiton) Smith is a well studied hybrid between *P. alba* and *P. tremula* [39]–[40], and *P.* × *jrtyschensis* Ch. Y. Yang is known to be a hybrid between *P. laurifolia* and *P. nigra* [41]–[42] according to morphological characters but has not previously been assessed genetically. For the 21 species used to evaluate the candidate barcode loci, 17 species were represented by two or more individuals (Table S1).

### Data Collection

Six candidate DNA barcodes, including two coding genes (*mat*K and *rbc*L) and three intergenic spacers (*trn*H-*psb*A, *trn*G-*psb*K and *psb*K-*psb*I) from the chloroplast genome, as well as a nuclear ITS region, were evaluated. Total DNA was extracted from silica-dried leaves using a modified cetyltrimethylammonium bromide (CTAB) method [43]. Polymerase chain reaction (PCR) amplifications were carried out using the primers listed in Table S2, following the protocol of Schroeder et al. [11] and the China Plant BOL Group et al. [16]. For DNA sequencing, we used the service provided by BGI (Beijing, China), and all sequences reported in this study have been deposited in NCBI GenBank under accession numbers KC485082- KC485262 (Table S1).

### Data Analyses

Sequence alignments were performed using MUSCLE [44] and refined manually in MEGA 5 [45]. Insertions/deletions (Indels) and single nucleotide polymorphisms (SNPs) were identified by DnaSP version 5.0 [46]. To assess the effects of barcode combinations on species discrimination, all two- or three-locus combinations and the combination of each chloroplast region and ITS were evaluated; we took this approach because two or three-locus barcodes are often recommended in published studies, e.g. [47]–[48]. To evaluate species discrimination success, we applied two different methods, PWG-Distance and Tree-Building. The PWG-Distance method (simple pair-wise matching for DNA barcoding) recommended by the CBOL Plant Working Group [15] employs distances calculated from pair-wise alignments counting unambiguous base substitutions only, and pair-wise p-distances were calculated using PAUP* 4.0b10 [49] and we considered discrimination to be successful if the minimum uncorrected interspecific p-distance involving a species was larger than its maximum intraspecific distance. When using the Tree-Building method, a Neighbor-Joining tree was constructed in the program PAUP*4.0b10 [49] under the Kimura 2-parameter substitution model, and species were considered discriminated if all individuals of a species formed a monophyletic group (e.g. [16]).

## Results

### Sequence Characterization

All the tested chloroplast regions, *mat*K, *rbc*L, *trn*H-*psb*A, *trn*G-*psb*K and *psb*K-*psb*I were successfully amplified with primers used in previous studies (Table S2). However, problems were encountered in the sequencing of *trn*H–*psb*A, where a mono-nucleotide repeat (poly(A)) fragment in the middle part of this region lead to sequencing failure of the latter half of this region for most individuals. Therefore this relatively short chloroplast region was sequenced with both forward and reverse primers and then assembled. Similar problems were also found in *psb*K-*psb*I for several individuals. When amplifying and sequencing the nuclear ITS region with universal primer combinations, about fifteen percent of the resultant sequences are unreadable, and several high quality sequences were very difficult to align with ITS sequences of *Populus*. These sequences were therefore examined using NCBI BLAST online, and the results showed that they are ITS regions belonging to fungi that occur on leaves of poplar (e.g. poplar crust). A *Populus* specific primer at the ITS1a end (Table S2) was then designed, and together with the universal primer at the ITS4 end, these fungal contaminated individuals were amplified and sequenced again. At last, chloroplast regions were successfully amplified and sequenced for all 21 species, while ITS sequences were successfully recovered for 20 species (all except *P*. *purdomii*).

In total, 555 new *Populus* sequences were generated in this study (Table S1). The aligned lengths of the six DNA regions range from 281 bp (*trn*H-*psb*A) to 966 bp (*rbc*L), and variable sites (SNPs) were lowest for *rbc*L (1.35%) and highest for ITS (6.54%) (Table 1). In addition, we found one and three heterozygous sites in the nuclear ITS sequences of the two putative hybrid species, *P. × canescens* and *P. × jrtyschensis*, respectively.

**Table 1:** Length, recovery rate, variation and delimitation rate of each DNA region and the combination of the five plastid regions.

Region	Seq. length (bp)	Recovery rate (%)	No. SNPs	%SNP	No. InDels	Rate (%) PWG	Rate (%) NJ
ITS	(568-)581	84.2	38	6.54	7	40.0	45.0
*mat*K	(766-)775	100	22	2.84	1	19.0	19.0
*trn*H-*psb*A	(281-)328	100	12	3.66	11	0.0	0.0
*psb*K-*psb*I	(480-)498	100	21	4.22	5	9.5	9.5
*rbc*L	966	100	13	1.35	0	0.0	0.0
*trn*G-*psb*K	(440-)462	100	17	3.68	7	9.5	9.5
Five cp regions	(2933-)3029	–	85	2.81	24	28.6	28.6

Seq. length: length of PCR product amplified with the given primers in bp; cp: chloroplast; No. SNPs: the number of SNPs; %SNP: percentage SNP calculated as the number of SNPs in relation to the longest sequence length; No. InDels: the number of Insertions/Deletions; Rate (%): percentage successful discrimination species calculated as the number of success discrimination species in relation to the total species; PWG: PWG-Distance method; NJ: Tree-Building method (Neighbor-Joining tree). Note that statistics for Seq. length, No. SNPs, %SNP and No. InDels were derived from an alignment of all (successfully sequenced) specimens.

### Species Discrimination

**Figure 1:**
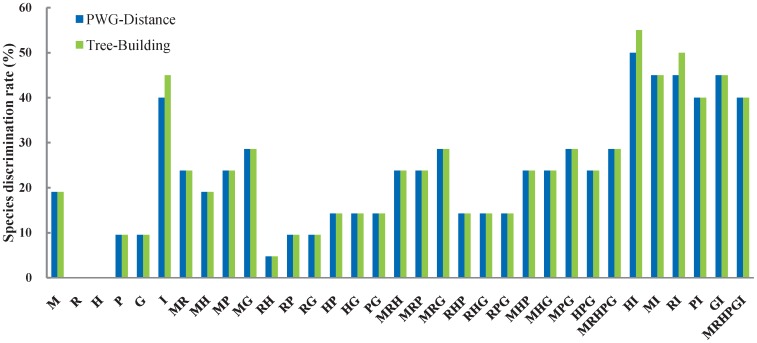
Species discrimination rate of all tested single- and multi- locus barcodes in *Populus*. M, *mat*K; R, *rbc*L; H, *trn*H-*psb*A; P, *psb*K-*psb*I; G, *trn*G-*psb*K; I, ITS; NJ, Neighbor-Joining.

Based on these sequences, we calculated the successful discrimination rate for six single regions and 27 combinations of two, three, five or six regions based on the PWG-Distance and Tree-Building methods (Fig. 1); note that the total number of species were 21 for any single chloroplast region or combination of chloroplast regions, and 20 for the nuclear ITS or combinations between this region and any single chloroplast region. In the following text, if there is one rate given for a single region or a combination of regions, this is the rate for both the PWG-Distance and Tree-Building methods. The discrimination efficacy ranged from 0 (*rbc*L, *trn*H-*psb*A) to 19.0% (*mat*K) for any single chloroplast locus, 4.8% (R+H) to 28.6% (M+G) for combinations of two chloroplast loci, 14.3% (R+H+P, R+H+G, R+P+G) to 28.6% (M+P+G, M+R+G) for combinations of three chloroplast loci; and was 28.6% for the combination of all five chloroplast loci (M+R+H+P+G). Meanwhile, the discrimination efficacy of the nuclear ITS region was 40.0% according to the PWG-Distance method and 45.0% according to the Tree-Building method; these values are higher than the efficacy of any single chloroplast locus or two- or three-locus combinations, and even higher than the combination of all chloroplast loci (Fig. 1). Furthermore, no chloroplast barcode was successful in differentiating the sister species, *P. euphratica* and *P. pruinosa*, in Section *Turanga* Bunge, but the nuclear ITS barcode was [33]. Among the five combinations of each chloroplast region and the nuclear ITS region, the discrimination efficacy was highest for I+H (Tree-Building: 55.0%, PWG-Distance: 50.0%), intermediate for I+R (Tree-Building: 50.0%, PWG-Distance: 45.0%), I+M (45%), and I+G (45%), and lowest for I+P (40%). At last, the combination of the nuclear ITS region and all five chloroplast regions successfully identified 8 out of 20 species (40%).

## Discussion

### Low Efficacy of the Five Chloroplast Locus Candidates

*Populus* is an economically and ecologically important genus of trees, and therefore considerable efforts have been put into the differentiation of widely cultivated species, especially cultivars and hybrids [10]–[11], [27], [29], [31]–[32], [50]. Until now, few studies have focused on the delimitation of wild *Populus* species (e.g. [11]), and the recent development of DNA barcoding provides a novel opportunity. DNA barcoding aims to identify species based on a single DNA region or a combination of a few DNA regions without taxonomic knowledge [12]. To achieve this, the barcode regions should be of short lengths, with high recovery rates (success rate for amplifying and sequencing) and have a high species discrimination rate [14]–[16], [48].

To date, most studies have examined chloroplast regions as candidates for plant barcoding because their faster mutation rate (compared to mitochondrial ones), non-recombination, uniparental inheritance and higher recovery rate (compared to nuclear ones) [14], [16], [51]. According to the CBOL Plant Working Group [15], their testing of seven chloroplast loci as barcoding candidates for 907 samples from 550 species, representing the major lineages of land plants, suggested that single locus, and two-, three-, and seven-locus combinations provided 43–69%, 59–75%, 65–76%, and 73% resolution at the species level, respectively. The China Plant BOL group et al. [16] undertook denser sampling (6286 samples representing 1757 species) and suggested a similar but slightly lower resolution (*rbc*L: 26.4%, *mat*K: 44.8%, *trn*H-*psb*A: 45.2%, *rbc*L+*mat*K: 49.7%; *rbc*L+*mat*K+*trn*H-*psb*A: 62.0%). Other large scale surveys of chloroplast regions as candidate barcodes, which involved fewer samples and species than the above two, have indicated similar or higher values for discriminate efficacy, e.g. [14], [52]–[53].

However, in this study, we found that all tested single chloroplast barcoding regions or different combinations of them (2-, 3- or 5-loci) have surprisingly low discrimination efficacy in *Populus* species from western China (Fig. 1). Among the single region barcodes, our highest discrimination rate was 19.0% for *matK*, while *rbc*L and *trn*H-*psb*A failed to differentiate any species (Fig. 1). For combinations of chloroplast loci, the highest discrimination rate was 28.6% for one 2-locus combination, two 3-locus combinations, as well as the combination of all five chloroplast regions. It is noteworthy that the core barcode region combination (*matK*+*rbcL*) recommended by the CBOL Plant Working Group [15] could only successfully identify 23.8% of all species, and adding *trn*H-*psb*A (M+R+H: 23.8%) did not improve the discrimination rate. This is in accordance with other investigations of potential candidate barcoding chloroplast regions within genera, which have revealed low discrimination rates for tree species, such as *Picea* (28.7% for a combination of seven chloroplast regions; [54]) and *Araucaria* (27.8% for a combination of five chloroplast regions; [48]).

### The Nuclear ITS Region is a more Promising Barcode in *Populus*

The nuclear ITS region has been suggested as barcode for plants by numerous authors (e.g. [14], [53], [55]–[56]), because this region evolves rapidly, leading to genetic changes that can differentiate closely related, congeneric species [14], [55], [57]. A recent test of this region across 1757 seed plant species further demonstrated its discrimination power at the species level [16]. In the current study, the successful delimitation rate for the nuclear ITS region is much higher (PWG-Distance: 40.0%; Tree-Building: 45.0%) than any single chloroplast region (≤19.0%) and even higher than the combination of all five chloroplast regions (28.6%) (Fig. 1).

These results suggest that, in *Populus*, the nuclear ITS region is a relatively effective barcode, out-competing chloroplast DNA regions. First, the nuclear ITS region is shorter and has a higher mutation rate than the chloroplast DNA regions (ITS: 6.54%, 38 variable sites out of 581 bp; five chloroplast markers combined: 2.81%, 85 variable sites out of 3029 bp) (Table 1). Secondly, compared to chloroplast DNA regions, the nuclear ITS region experiences less introgressions. Introgressions can lead to transfer of genetic material across species boundaries, and genomic components with lower intraspecific gene flow are prone to introgression [58]–[59]. In *Populus*, the nuclear ITS region is biparentally inherited and dispersed by both pollen and seeds, while the chloroplast DNA regions are maternally inherited and dispersed only by seeds [2]. Thus, the ITS region has higher intraspecific gene flow, lower interspecific gene flow (i.e. lower introgression) and higher inter-species differentiation than the chloroplast regions. Thirdly, the nuclear ITS regions in plants comprise multiple (reiterated) copies and usually experience concerted evolution [60]. During this process, these different copies become homogenized to the same sequence type (at least become almost identical type) as a result of mechanisms such as high-frequency unequal crossing over or gene conversion [60]. Therefore, this region may have undergone fast lineage sorting and subsequently interspecific differentiation, comparable even to that experienced by speciation genes and linked fragments [33], [60]. Considered together, the characteristics of plant nuclear ITS regions, such as high mutation rate, low introgression and concerted evolution, have facilitated higher species delimitation efficiency of this nuclear marker than chloroplast markers in *Populus*.

### A Combined Nuclear and Chloroplast Barcode is an Effective Approach in Species Delimitation and Hybridization Detection

Since the nuclear ITS region and the chloroplast regions have different inheritance modes and track different evolutionary histories, a combination of them will improve species delimitation power and further our understanding of evolutionary processes in plants [16]. In this study, we found that among *Populus* species from western China, using a combination of the ITS region and a single chloroplast region usually improved discrimination efficiency (Fig. 1). This is in agreement with previous work on DNA barcoding of single plant genera, e.g. *Alnus* (Betulaceae) [61] and *Holcoglossum* (Orchidaceae) [62].

Among the five combinations of ITS region and single chloroplast region, the combination H+I differentiated 11/10 species out of 20 (Tree-Building: 55.0%, PWG-Distance: 50.0%), while the combinations R+I, G+I, M+I and P+I differentiated 10/9 (Tree-Building: 50%, PWG-Distance: 45%), 9/9 (45%), 9/9 (45%), 8/8 (40%) species out of 20, respectively. These values are higher than achieved by any of these five chloroplast regions individually (0.0%–19.0%), and are higher than or similar to those derived from the nuclear ITS (Tree-Building: 45.0%, PWG-Distance: 40.0%).

**Figure 2:**
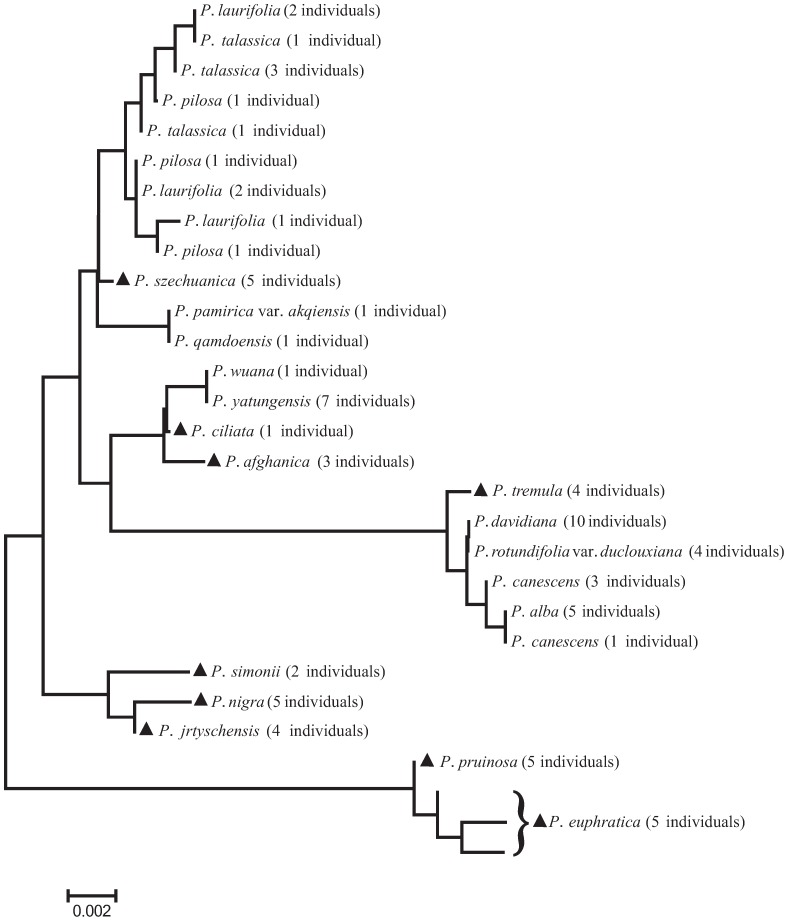
Neighbor-Joining (NJ) tree based on the nuclear internal transcribed spacer (ITS) region. Species with solid triangles were successfully delimited using the Tree-Building method.

**Figure 3:**
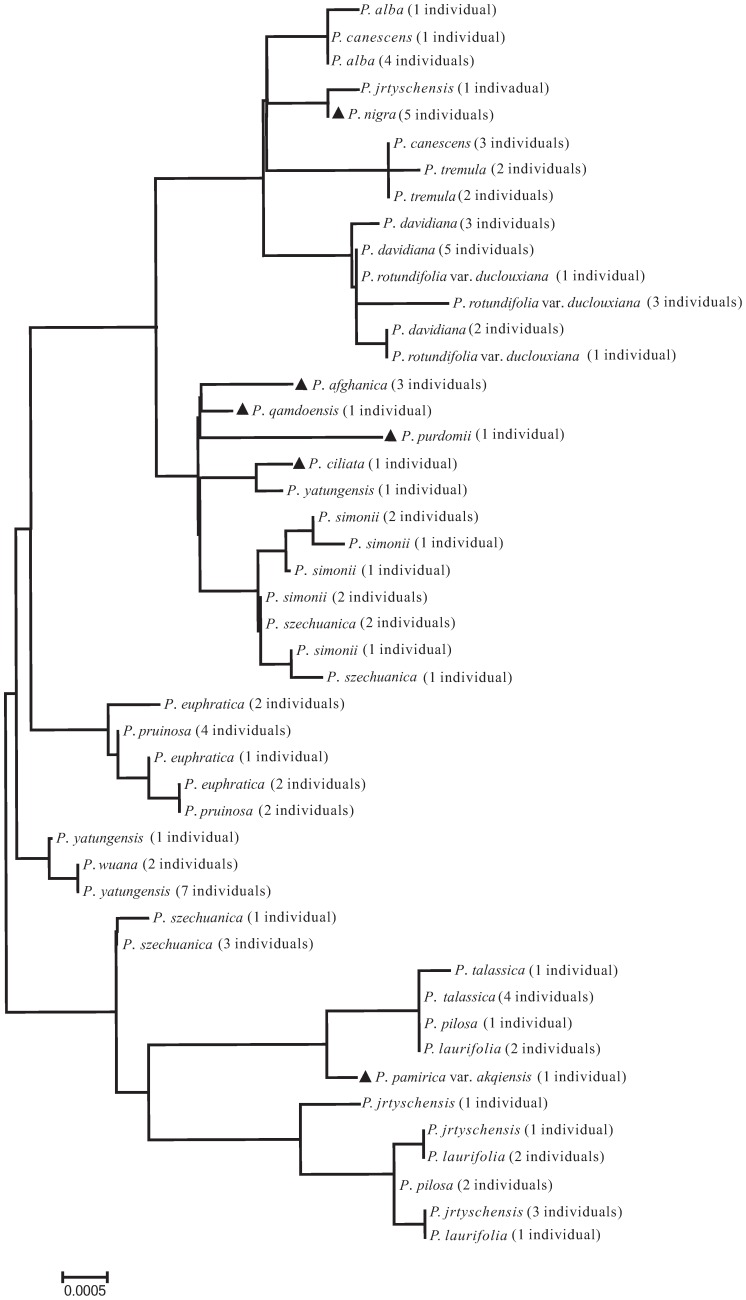
Neighbor-Joining (NJ) tree based on the combination of all five chloroplast regions. Species with solid triangles were successfully delimited using the Tree-Building method.

Meanwhile, the combinations of nuclear and chloroplast regions are also useful in understanding the evolutionary process experienced by plants, e.g. detecting recent hybridization events. There are two such cases in our study. *P. × canescens* is a cross between *P. alba* and *P. tremula*, and its parentage has been clearly demonstrated in previous studies (e.g. [39]–[40]), and our study confirmed that *P. × canescens* individuals from Xinjiang province are also hybrids between the above two parental species (Figs. 2, 3); however, ITS sequences of these individuals are closer to *P. tremula* (Fig. 2). Another case is *P. × jrtyschensis*, which is generally thought to be the natural hybrid between *P. nigra* and *P. laurifolia* [41]–[42], since the morphology of *P. × jrtyschensis* obviously combines characters from both putative parent species. However, this hypothesis, based on morphological characters, needs to be tested further and, indeed, our results provided positive evidence for the assumed parentage. On one hand, ITS sequences of *P. × jrtyschensis* have three sites that combined SNPs from the *P. nigra* and *P. laurifolia* complex, which includes *P. laurifolia*, *P. talassica* and *P. pilosa* (Figs. 2, 3), although the Tree-Building method revealed that ITS genotypes of all hybrid individuals from different populations are closer to *P. nigra* (Fig. 2). On the other hand, the chloroplast haplotype of hybrid individuals clustered with either the *P. laurifolia* complex or *P. nigra* (Fig. 3), which suggests that there are hybrid individuals with either *P. nigra* or the *P. laurifolia* complex as maternal donor species. As shown above, the combination of the nuclear ITS region and chloroplast region is an effective approach for detecting recent hybridization events.

### Improving the Taxonomy and DNA Barcoding System in Wild *Populus* Species

Although *Populus* species are widely distributed across the northern hemisphere, most research concerning this genus has focused on the six to eight species that have been widely cultivated and/or commercially exploited, i.e. *P. alba*, *P. tremula*, *P. tremuloides*, *P. nigra*, *P. deltoides*, *P. trichocarpa* (ref. [11] and references therein). As yet, there is no consensus on the true number of *Populus* species globally due to misinterpretation of hybrids and difficulties in delimitating species boundaries. Frequent interspecific hybridizations and introgressions as well as a high level of intraspecific morphological variation in this genus are the main causes (e.g. [2], [9], [38]). Previous studies have suggested that *Populus* comprises 22 to 85 species plus hundreds of hybrids, varieties and cultivars [2], [3], [9], [63]. Although the widely accepted taxonomic treatment [9] classified *Populus* into 29 species in six sections (*Abaso*, *Aigeiros*, *Leucoides*, *Populus*, *Tacamahaca*, *Turanga*), the Flora of China [64] recognized 71 species (47 endemic, including at least nine hybrids) from five sections (all sections except *Abaso*). Given the sharp contrast between different authors with respect to the number of recognized species in this economically and ecologically important genus, it is imperative to initiate a large scale taxonomic revision of *Populus* across the world, especially in China. DNA barcoding may be an appropriate tool for this, as it is considered to represent a powerful methodology in testing existing taxonomic treatments based solely on morphological characters (e.g. [61], [65]).

In this study, we undertook a study of the DNA barcoding of 21 species from western China, employing the nuclear ITS region and five chloroplast regions (*mat*K, *rbc*L, *trn*H-*psb*A, *trn*G-*psb*K and *psb*K-*psb*I). As discussed above, each of these markers and their combinations have different success rates in discriminating species. Their discrimination rates may vary as a result of many factors, including mutation rate and inheritance mode. However, discriminating between closely related *Populus* species from parapatric or sympatric areas in western China using DNA barcodes always failed. This may have been the result of incomplete lineage sorting, interspecific hybridization and/or introgressions [59], [66]. In order to delimit species boundaries between these sympatric and parapatric species, as well as species boundaries for widespread species that often encounter local species, studies based on molecular data (e.g. DNA sequence, microsatellite, AFLP etc.) at population level are essential (e.g. [33]).

Therefore, we agree with a previous proposal for the improvement of DNA barcoding success rate, namely the introduction of highly variable loci that are nested under core barcoding markers, i.e. build a tiered barcoding system [67]. Such a barcoding system would be especially valuable for economically and ecologically important genera, such as *Populus*. Based on our results, we suggest that in *Populus*, species delimitation should be performed at least at two levels: the inter-species-complex level and the intra-species-complex level. First, performing a large scale survey of species or species complex (a group of close related species in which gene flows occur frequently) boundaries among all wild species from as many populations as possible using moderately variable loci. Here we recommend the combination of the nuclear marker ITS and one (*trn*H-*psb*A) chloroplast maker, and the combination of two (*mat*K and *trn*G-*psb*K) or three chloroplast markers (*mat*K, *psb*K-*psb*I and *trn*G-*psb*K). Subsequently, screening should focus on each species complex, based on population genetics studies using highly variable loci and a denser sampling strategy. Although such studies at population level are usually performed in order to infer the evolutionary history and population genetics of each species complex (e.g. divergence and gene flow among species, demographic history of each species etc.), they are also helpful in detecting differentiation between species [33], [68]. Two types of molecular markers with stable recovery rates and easy sample preparation, nuclear SNPs and nuclear SSRs, have particular potential in such studies. Previous studies suggest that Nuclear SNPs is able to identify widely cultivated *Populus* species [10], [32], and the combinations of nuclear SSRs are able to identify different *Populus* species [27], [29], [31], [33]. The development of nuclear SNP markers and nuclear SSRs (and multiplex SSRs, e.g. [69]) became increasingly convenient given the sequencing of genome (e.g. [8]) and transcriptome (e.g. [70]–[71]) of *Populus* species as well as re-sequencing at the population level (e.g. [72]).

In conclusion, we consider that the current taxonomy of wild *Populus* species needs further revisions with the help of a tiered DNA barcoding system, where different DNA barcodes are applied at inter- and intra-species-complex levels. Doubtless, a tiered DNA barcoding system is extremely important in taxonomic groups that have experienced a complex evolutionary history, e.g. with frequent hybridizations and introgressions among species.

## Supporting Information

Figure S1 **The heterozygous sites in the nuclear ITS sequences of** ***P.×jrtyschensis*** **(upper) and** ***P.×canescens*** **(lower).**
(DOCX)

Table S1 **Provenance of samples and GenBank accession numbers.**
(DOCX)

Table S2 **Primers used for amplification and sequencing.**
(DOCX)

## References

[1] Stettler RF, Bradshaw T, Heilman P, Hinckley T (1996) Biology of *Populus* and its implications for management and conservation. Montreal, Canada: NRC Research Press.

[2] Hamzeh M, Dayanandan S (2004) Phylogeny of *Populus* (Salicaceae) based on nucleotide sequences of chloroplast *trn*T-*trn*F region and nuclear rDNA. American Journal of Botany 91: 1398–1408. 21652373 10.3732/ajb.91.9.1398

[3] Dickman DI, Stuart K (1983) The culture of poplars in Eastern North America. Department of Forestry, Michigan State University, East Lansing.

[4] Heilman PE (1999) Planted forests: poplars. New Forests 17: 89–93.

[5] Bradshaw HD, Ceulemans R, Davis J, Stettler R (2000) Emerging Model Systems in Plant Biology: Poplar (*Populus*) as A Model Forest Tree. J Plant Growth Regul 19: 306–313.

[6] Jansson S, Douglas CJ (2007) *Populus*: a model system for plant biology. Annu Rev Plant Biol 58: 435–458. 17280524 10.1146/annurev.arplant.58.032806.103956

[7] Ellis B, Jansson S, Strauss SH, Tuskan GA (2010) Why and How *Populus* Became a “Model Tree”. In: Jansson S, Bhalerao R, Groover A, editors. Genetics and Genomics of *Populus*. New York: Springer. 3–14.

[8] Tuskan GA, Difazio S, Jansson S, Bohlmann J, Grigoriev I, et al (2006) The genome of black cottonwood, *Populus trichocarpa* (Torr. & Gray). Science 313: 1596–1604. 16973872 10.1126/science.1128691

[9] Eckenwalder JE (1996) Systematics and evolution of *Populus* In: Stettler RF, Bradshaw HD, Heilman PE, Hinckley TM, editors. Biology of *Populus* and its implications for management and conservation. Montreal, Canada: NRC Research Press. 7–32.

[10] Fladung M, Buschbom J (2009) Identification of single nucleotide polymorphisms in different *Populus* species. Trees Struct Funct 23: 1199–1212.

[11] Schroeder H, Hoeltken AM, Fladung M (2012) Differentiation of *Populus* species using chloroplast single nucleotide polymorphism (SNP) markers–essential for comprehensible and reliable poplar breeding. Plant Biol (Stuttg) 14: 374–381. 21973311 10.1111/j.1438-8677.2011.00502.x

[12] Hebert PDN, Cywinska A, Ball SL, deWaard JR (2003) Biological identifications through DNA barcodes. Proc Biol Sci 270: 313–321. 12614582 10.1098/rspb.2002.2218 PMC1691236

[13] Hebert PDN, Penton EH, Burns JM, Janzen DH, Hallwachs W (2004) Ten species in one: DNA barcoding reveals cryptic species in the neotropical skipper butterfly *Astraptes fulgerator* . Proc Natl Acad Sci USA 101: 14812–14817. 15465915 10.1073/pnas.0406166101 PMC522015

[14] Kress WJ, Wurdack KJ, Zimmer EA, Weigt LA, Janzen DH (2005) Use of DNA barcodes to identify flowering plants. Proc Natl Acad Sci USA 102: 8369–8374. 15928076 10.1073/pnas.0503123102 PMC1142120

[15] CBOL Plant Working Group (2009) A DNA barcode for land plants. Proc Natl Acad Sci USA 106: 12794–12797. 19666622 10.1073/pnas.0905845106 PMC2722355

[16] China Plant BOL Group, Li DZ, Gao LM, Li HT, Wang H, et al (2011) Comparative analysis of a large dataset indicates that internal transcribed spacer (ITS) should be incorporated into the core barcode for seed plants. Proc Natl Acad Sci USA 108: 19641–19646. 22100737 10.1073/pnas.1104551108 PMC3241788

[17] Vences M, Thomas M, Bonett RM, Vieites DR (2005) Deciphering amphibian diversity through DNA barcoding: chances and challenges. Phil Trans R Soc B 360: 1859–1868. 16221604 10.1098/rstb.2005.1717 PMC1609216

[18] Hebert PDN, Stoeckle MY, Zemlak TS, Francis CM (2004) Identification of birds through DNA Barcodes. PLoS Biol 2: e312. 15455034 10.1371/journal.pbio.0020312 PMC518999

[19] Ward RD, Hanner R, Hebert PDN (2009) The campaign to DNA barcode all fishes, FISH-BOL. J Fish Biol 74: 329–356. 20735564 10.1111/j.1095-8649.2008.02080.x

[20] Cho Y, Qiu YL, Kuhlman P, Palmer JD (1998) Explosive invasion of plant mitochondria by a group I intron. Proc Natl Acad Sci USA 95: 14244–14249. 9826685 10.1073/pnas.95.24.14244 PMC24358

[21] Cho Y, Mower JP, Qiu YL, Palmer JD (2004) Mitochondrial substitution rates are extraordinarily elevated and variable in a genus of flowering plants. Proc Natl Acad Sci USA 101: 17741–17746. 15598738 10.1073/pnas.0408302101 PMC539783

[22] Cowan RS, Chase MW, Kress WJ, Savolainen V (2006) 300,000 species to identify: problems, progress, and prospects in DNA barcoding of land plants. Taxon 55: 611–616.

[23] Pennisi E (2007) Taxonomy. Wanted: a barcode for plants. Science 318: 190–191. 17932267 10.1126/science.318.5848.190

[24] Hollingsworth PM (2011) Refining the DNA barcode for land plants. Proc Natl Acad Sci USA 108: 19451–19452. 22109553 10.1073/pnas.1116812108 PMC3241790

[25] Rajora OP (1990) Marker allozyme genes and alleles for differentiation of *Populus deltoides*, *P. nigra*, *P. maximowiczii*, and their interspecific hybrids. Can J Bot 68: 990–998.

[26] Benetka V, Mottl J, Vackova K, Pospiskova M, Dubsky M (1999) Estimation of the introgression level in *Populus nigra* L. populations by means of isozyme gene markers. Silvae Genetica 48: 218–223.

[27] Rahman MH, Rajora OP (2002) Microsatellite DNA fingerprinting, differentiation, and genetic relationships of clones, cultivars, and varieties of six poplar species from three sections of the genus *Populus* . Genome 45: 1083–1094. 12502253 10.1139/g02-077

[28] Cervera MT, Storme V, Soto A, Ivens B, Montagu MV, et al (2005) Intraspecific and interspecific genetic and phylogenetic relationships in the genus *Populus* based on AFLP markers. Theor Appl Genet 111: 1440–1456. 16211377 10.1007/s00122-005-0076-2

[29] Fossati T, Zapelli I, Bisoffi S, Micheletti A, Vietto L, et al (2005) Genetic relationships and clonal identity in a collection of commercially relevant poplar cultivars assessed by AFLP and SSR. Tree Gene Gen 1: 11–20.

[30] De-Lucas AI, Santana JC, Recio P, Hidalgo E (2008) SSR-based tool for identification and certification of commercial *Populus* clones in Spain. Ann Forest Sci 65: 107.

[31] Liesebach H, Schneck V, Ewald E (2010) Clonal fingerprinting in the genus *Populus* L. by nuclear microsatellite loci regarding differences between sections, species and hybrids. Tree Gene Gen 6: 259–269.

[32] Schroeder H, Fladung M (2010) SSR and SNP Markers for the Identification of Clones, Hybrids and Species Within the Genus *Populus* . Silvae Genetica 59: 257–263.

[33] Wang J, Wu Y, Ren G, Guo Q, Liu J, et al (2011) Genetic Differentiation and Delimitation between Ecologically Diverged *Populus euphratica* and *P. pruinosa* . PLoS ONE 6: e26530. 22028897 10.1371/journal.pone.0026530 PMC3197521

[34] Hamzeh M, Sawchyn C, Périnet P, Dayanandan S (2007) Asymmetrical natural hybridization between *Populus deltoides* and *P. balsamifera* (Salicaceae). Can J Bot 85: 1227–1232.

[35] Stölting KN, Nipper R, Lindtke D, Caseys C, Waeber S, et al (2013) Genomic scan for single nucleotide polymorphisms reveals patterns of divergence and gene flow between ecologically divergent species. Mol Ecol 22: 842–855. 22967258 10.1111/mec.12011

[36] Vanden Broeck A, Storme V, Cottrell JE, Boerjan W, Van Bockstaele E, et al (2004) Gene flow between cultivated poplars and native black poplar (*Populus nigra* L.): a case study along the river Meuse on the Dutch-Belgian border. Forest Ecol Manag 197: 307–310.

[37] Meirmans PG, Lamothe M, Gros-Louis M-C, Khasa D, Périnet P, et al (2010) Complex patterns of hybridization between exotic and native North American poplar species. Am J Bot 97: 1688–1697. 21616802 10.3732/ajb.0900271

[38] Thompson SL, Lamothe M, Meirmans PG, PÉRinet P, Isabel N (2010) Repeated unidirectional introgression towards *Populus balsamifera* in contact zones of exotic and native poplars. Mol Ecol 19: 132–145. 10.1111/j.1365-294X.2009.04442.x 20002578

[39] Van Loo M, Joseph JA, Heinze B, Fay MF, Lexer C (2008) Clonality and spatial genetic structure in *Populus* × *canescens* and its sympatric backcross parent P. *alba* in a Central European hybrid zone. New Phytol 177: 506–516. 18005320 10.1111/j.1469-8137.2007.02266.x

[40] He C, Zheng S, Zhang J, Duan A, Zeng Y, et al (2010) Clonal reproduction and natural variation of *Populus canescens* patches. Tree Physiol 30: 1383–1390. 21030405 10.1093/treephys/tpq083

[41] Wang C, Fang CF, Zhao SD, Chou YL, Tung SL, et al. (1984) *Populus* L. In: Wang C, Fang CF, editors. Flora Republicae Popularis Sinicae Tomus 20(2). Beijing: Science Press. 1–78.

[42] Yang CY, Shen KM, Mao ZM (1992) *Populus* L. In: Yang CY, editor. Flora Xinjiangensis Tomus 1. Urumqi Xinjiang Science, Technology & Hygiene Publishing House. 122–158.

[43] Doyle JJ, Doyle JL (1987) A rapid DNA isolation procedure for small quantities of fresh leaf tissue. Phytochem Bull 19: 11–15.

[44] Edgar RC (2004) MUSCLE: multiple sequence alignment with high accuracy and high throughput. Nucleic Acids Res 32: 1792–1797. 15034147 10.1093/nar/gkh340 PMC390337

[45] Tamura K, Peterson D, Peterson N, Stecher G, Nei M, et al (2011) MEGA5: molecular evolutionary genetics analysis using maximum likelihood, evolutionary distance, and maximum parsimony methods. Mol Biol Evol 28: 2731–2739. 21546353 10.1093/molbev/msr121 PMC3203626

[46] Librado P, Rozas J (2009) DnaSP v5: a software for comprehensive analysis of DNA polymorphism data. Bioinformatics 25: 1451–1452. 19346325 10.1093/bioinformatics/btp187

[47] Fazekas AJ, Burgess KS, Kesanakurti PR, Graham SW, Newmaster SG, et al (2008) Multiple multilocus DNA barcodes from the plastid genome discriminate plant species equally well. PLoS ONE 3: e2802. 18665273 10.1371/journal.pone.0002802 PMC2475660

[48] Hollingsworth ML, Andra Clark A, Forrest LL, Richardson J, Pennington RT, et al (2009) Selecting barcoding loci for plants: evaluation of seven candidate loci with species-level sampling in three divergent groups of land plants. Mol Ecol Resour 9: 439–457. 21564673 10.1111/j.1755-0998.2008.02439.x

[49] Swofford DL (2002) PAUP*: phylogenetic analyses using parsimony (*and other methods), Version 4.: Sinauer Associates, Sunderland, MA.

[50] Meirmans PG, Lamothe M, Perinet P, Isabel N (2007) Species-specific single nucleotide polymorphism markers for detecting hybridization and introgression in poplar. Can J Bot 85: 1082–1091.

[51] Chase MW, Salamin N, Wilkinson M, Dunwell JM, Kesanakurthi RP, et al (2005) Land plants and DNA barcodes: short-term and long-term goals. Philos Trans R Soc Lond B Biol Sci 360: 1889–1895. 16214746 10.1098/rstb.2005.1720 PMC1609218

[52] Kress WJ, Erickson DL (2007) A two-locus global DNA barcode for land plants: the coding *rbc*L gene complements the non-coding *trn*H-*psb*A spacer region. PLoS ONE 2: e508. 17551588 10.1371/journal.pone.0000508 PMC1876818

[53] Chen SL, Yao H, Han JP, Liu C, Song JY, et al (2010) Validation of the ITS2 Region as a Novel DNA Barcode for Identifying Medicinal Plant Species. PLoS ONE 5: e8613. 20062805 10.1371/journal.pone.0008613 PMC2799520

[54] Ran JH, Wang PP, Zhao HJ, Wang XQ (2010) A Test of Seven Candidate Barcode Regions from the Plastome in *Picea* (Pinaceae). J Integr Plant Biol 52: 1109–1126. 21106009 10.1111/j.1744-7909.2010.00995.x

[55] Sass C, Little DP, Stevenson DW, Specht CD (2007) DNA Barcoding in the Cycadales: Testing the Potential of Proposed Barcoding Markers for Species Identification of Cycads. PLoS ONE 2: e1154. 17987130 10.1371/journal.pone.0001154 PMC2063462

[56] Yao H, Song JY, Liu C, Luo K, Han JP, et al (2010) Use of ITS2 Region as the Universal DNA Barcode for Plants and Animals. PLoS ONE 5: e13102. 20957043 10.1371/journal.pone.0013102 PMC2948509

[57] Nieto Feliner G, Rossello JA (2007) Better the devil you know? Guidelines for insightful utilization of nrDNA ITS in species-level evolutionary studies in plants. Mol Phylogenet Evol 44: 911–919. 17383902 10.1016/j.ympev.2007.01.013

[58] Du FK, Petit RJ, Liu JQ (2009) More introgression with less gene flow: chloroplast vs. mitochondrial DNA in the *Picea asperata* complex in China, and comparison with other Conifers. Mol Ecol 18: 1396–1407. 19284474 10.1111/j.1365-294X.2009.04107.x

[59] Petit RJ, Excoffier L (2009) Gene flow and species delimitation. Trends Ecol Evol 24: 386–393. 19409650 10.1016/j.tree.2009.02.011

[60] Alvarez I, Wendel JF (2003) Ribosomal ITS sequences and plant phylogenetic inference. Mol Phylogenet Evol 29: 417–434. 14615184 10.1016/s1055-7903(03)00208-2

[61] Ren BQ, Xiang XG, Chen ZD (2010) Species identification of *Alnus* (Betulaceae) using nrDNA and cpDNA genetic markers. Mol Ecol Resour 10: 594–605. 21565064 10.1111/j.1755-0998.2009.02815.x

[62] Xiang XG, Hu H, Wang W, Jin XH (2011) DNA barcoding of the recently evolved genus *Holcoglossum* (Orchidaceae: Aeridinae): a test of DNA barcode candidates. Mol Ecol Resour 11: 1012–1021. 21722327 10.1111/j.1755-0998.2011.03044.x

[63] Eckenwalder JE (1977) Systematics of *Populus* L. in southwestern North America with special reference to sect. *Aigeiros* Duby. Berkeley, California, USA: University of California.

[64] Fang CF, Zhao SD, Skvortsov AK (1999) Salicaceae Mirbel: 1. *Populus* Linnaeus. In: Wu CY, Raven PH, editors. Flora of China Vol 4: Beijing: Science Press & St. Louis: Missouri Botanical Garden Press. 139–162.

[65] Hajibabaei M, Singer GAC, Hebert PDN, Hickey DA (2007) DNA barcoding: how it complements taxonomy, molecular phylogenetics and population genetics. Trends Genet 23: 167–172. 17316886 10.1016/j.tig.2007.02.001

[66] Hollingsworth PM, Graham SW, Little DP (2011) Choosing and using a plant DNA barcode. PLoS ONE6: e19254. 10.1371/journal.pone.0019254 PMC3102656 21637336

[67] Newmaster SG, Fazekas AJ, Ragupathy S (2006) DNA barcoding in land plants: evaluation of *rbc*L in a multigene tiered approach. Can J Bot 84: 335–341.

[68] Lexer C, Fay MF, Joseph JA, Nica MS, Heinze B (2005) Barrier to gene flow between two ecologically divergent *Populus* species, *P. alba* (white poplar) and *P. tremula* (European aspen): the role of ecology and life history in gene introgression. Mol Ecol 14: 1045–1057. 15773935 10.1111/j.1365-294X.2005.02469.x

[69] Xu F, Feng S, Wu R, Du FK (2013) Two highly validated SSR multiplexes (8-plex) for Euphrates’ poplar, *Populus euphratica* (Salicaceae). Mol Ecol Resour 13: 144–153. 23134475 10.1111/1755-0998.12030

[70] Qiu Q, Ma T, Hu QJ, Liu BB, Wu YX, et al (2011) Genome-scale transcriptome analysis of the desert poplar, *Populus euphratica* . Tree Physiol 31: 452–461. 21427158 10.1093/treephys/tpr015

[71] Du FK, Xu F, Qu H, Feng S, Tang J, et al (2013) Exploiting the transcriptome of Euphrates Poplar, *Populus euphratica* (Salicaceae) to develop and characterize new EST-SSR markers and construct an EST-SSR database. PLoS ONE 8: e61337. 23593466 10.1371/journal.pone.0061337 PMC3623821

[72] Slavov GT, DiFazio SP, Martin J, Schackwitz W, Muchero W, et al (2012) Genome resequencing reveals multiscale geographic structure and extensive linkage disequilibrium in the forest tree *Populus trichocarpa* . New Phytol 196: 713–725. 22861491 10.1111/j.1469-8137.2012.04258.x

