# Supplementary material for: Barcoding Poplars (Populus L.) from Western China
Source: PLoS One. 2013 Aug 19;8(8):e71710. doi: 10.1371/journal.pone.0071710 (PMC3747233; doi:10.1371/journal.pone.0071710)
Supplement: Figure S1 — The heterozygous sites in the nuclear ITS sequences of P.×jrtyschensis (upper) and P.×canescens (lower). (DOCX) [file pone.0071710.s001.docx]

Figure S1. The heterozygous sites in the nuclear ITS sequences of *P. × jrtyschensis* (upper) and *P. × canescens* (lower).
