# Supplementary material for: Barcoding Poplars (Populus L.) from Western China
Source: PLoS One. 2013 Aug 19;8(8):e71710. doi: 10.1371/journal.pone.0071710 (PMC3747233; doi:10.1371/journal.pone.0071710)
Supplement: Table S1 — Provenance of samples and GenBank accession numbers. (DOCX) [file pone.0071710.s002.docx]

Table S1．Provenance of samples

| Taxa | Sources/individuals/vouchers | GenBank accession numbers | | | | | |
| --- | --- | --- | --- | --- | --- | --- | --- |
|  |  | *ITS* | *mat*K | *psb*A*-trn*H | *psb*K*-psb*I | *rbc*L | *trnG-psbK* |
| Section *Populus* |  |  |  |  |  |  |  |
| *Populus alba* var. *alba* L. | Beitun, Xinjiang,China/1/ LiuJQ-XJ-2011-027 | KC485083 | KC485112 | KC485141 | KC485171 | KC485204 | KC485230 |
|  | Fuhai, Xinjiang,China/1/LiuJQ-XJ-2011-032 | KC485083 | KC485112 | KC485141 | KC485171 | KC485204 | KC485230 |
|  | Habahe, Xinjiang,China/1/LiuJQ-XJ-2011-061 | KC485083 | KC485112 | KC485141 | KC485171 | KC485204 | KC485230 |
|  | 185tuan, Xinjiang,China/1/LiuJQ-XJ-2011-069 | KC485083 | KC485112 | KC485141 | KC485171 | KC485204 | KC485230 |
|  | Habahe, Xinjiang,China/1/LiuJQ-XJ-2011-074 | KC485083 | KC485112 | KC485141 | KC485171 | KC485205 | KC485230 |
| *P. × canescens* (Aiton) Simth | Habahe, Xinjiang,China/1/LiuJQ-XJ-2011-062 | KC485084 | KC485113 | KC485142 | KC485172 | KC485206 | KC485231 |
|  | Burqin, Xinjiang,China/1/LiuJQ-XJ-2011-064 | KC485085 | KC485113 | KC485142 | KC485172 | KC485206 | KC485231 |
|  | 185tuan, Xinjiang,China/1/LiuJQ-XJ-2011-068 | KC485085 | KC485113 | KC485143 | KC485172 | KC485206 | KC485232 |
|  | 185tuan, Xinjiang,China/1/LiuJQ-XJ-2011-072 | KC485085 | KC485113 | KC485142 | KC485172 | KC485206 | KC485231 |
| *P. davidiana* Dode. | Diebu,Gansu, china/1/ RY008 | KC485087 | KC485115 | KC485146 | KC485174 | KC485208 | KC485234 |
|  | Tongren,qinghai, china/1/RY036 | KC485087 | KC485115 | KC485146 | KC485174 | KC485208 | KC485235 |
|  | Xunhua, qinghai, china/1/RY038 | KC485087 | KC485115 | KC485146 | KC485174 | KC485208 | KC485235 |
|  | Minhe, qinghai, china/1/RY048 | KC485087 | KC485115 | KC485146 | KC485174 | KC485208 | KC485234 |
|  | Huangyuan, qinghai, china/1/RY054 | KC485087 | KC485115 | KC485146 | KC485174 | KC485208 | KC485234 |
|  | Huzhu, qinghai, china/1/RY060 | KC485087 | KC485115 | KC485146 | KC485174 | KC485208 | KC485234 |
|  | Linzhi, Xizang,China/1/LiuJQ-QTP-2011-159 | KC485087 | KC485115 | KC485145 | KC485174 | KC485208 | KC485234 |
|  | Milin, Xizang,China/1/LiuJQ-QTP-2011-165 | KC485087 | KC485115 | KC485145 | KC485174 | KC485208 | KC485234 |
|  | Markang,Sichuan,China/1/LiuJQ-QTP-2011-270 | KC485087 | KC485115 | KC485146 | KC485174 | KC485208 | KC485235 |
|  | Markang, Sichuan,China/1/LiuJQ-QTP-2011-273 | KC485087 | KC485115 | KC485146 | KC485174 | KC485208 | KC485234 |
| *P. tremula* L. | Fuyun, Xinjiang,China/1/LiuJQ-XJ-2011-015 | KC485108 | KC485133 | KC485166 | KC485198 | KC485222 | KC485257 |
|  | Fuyun, Xinjiang,China/1/LiuJQ-XJ-2011-019 | KC485108 | KC485133 | KC485166 | KC485198 | KC485222 | KC485258 |

Continued

Table 1．Continued

| Taxa | Sources/individuals/vouchers | GenBank accession numbers | | | | | |
| --- | --- | --- | --- | --- | --- | --- | --- |
|  |  | *ITS* | *mat*K | *psb*A*-trn*H | *psb*K*-psb*I | *rbc*L | *trnG-psbK* |
|  | Altay, Xinjiang,China/1/LiuJQ-XJ-2011-038 | KC485108 | KC485133 | KC485166 | KC485198 | KC485222 | KC485257 |
|  | Habahe, Xinjiang,China/1/LiuJQ-XJ-2011-063 | KC485108 | KC485133 | KC485166 | KC485198 | KC485222 | KC485258 |
| *P. rotundifolia* Griff. var. *duclouxiana* (Dode) Gomb. | Gongbujiangda, Xizang,China/1/LiuJQ-QTP-2011-151 | KC485102 | KC485125 | KC485159 | KC485191 | KC485218 | KC485249 |
|  | Langxian, Xizang,China/1/LiuJQ-QTP-2011-173 | KC485102 | KC485126 | KC485160 | KC485191 | KC485218 | KC485249 |
|  | Zhamu, Xizang,China/1/LiuJQ-QTP-2011-199 | - | KC485126 | KC485160 | KC485191 | KC485218 | KC485249 |
|  | Changdu, Xizang,China/1/LiuJQ-QTP-2011-203 | KC485102 | KC485126 | KC485160 | KC485191 | KC485218 | KC485249 |
|  | Ruoergai, Sichuan,China/1/LiuJQ-QTP-2011-291 | KC485102 | KC485125 | KC485159 | KC485191 | KC485218 | KC485249 |
| *P.* *purdomii* Rehd. | Zhada, Xizang,China/1/LliuJQ-QTP-2011-067 | - | KC485134 | KC485167 | KC485199 | KC485223 | KC485259 |
| Sect. *Tacamahaca* Spach |  |  |  |  |  |  |  |
| *P. simonii* Carr. | Diebu,Gansu, china/1/RY006 | - | KC485127 | KC485161 | KC485192 | KC485219 | KC485251 |
|  | Huzhu, qinghai, china/1/RY063 | KC485103 | KC485128 | KC485162 | KC485192 | KC485219 | KC485251 |
|  | Qilian, qinghai, china/1/RY076 | KC485103 | KC485128 | KC485161 | KC485192 | KC485219 | KC485251 |
|  | Diebu,Gansu, China/1/LiuJQ-QTP-2011-290 | - | KC485127 | KC485161 | KC485192 | KC485219 | KC485250 |
|  | Danba,Sichuan, China/1/ LiuJQ-YW-2012-050 | - | KC485127 | KC485161 | KC485192 | KC485219 | KC485252 |
|  | Jinchuan,Sichuan, China/1/ LiuJQ-YW-2012-056 | - | KC485127 | KC485161 | KC485192 | KC485219 | KC485252 |
|  | Minhe,Qinghai, China/1/ LiuJQ-YW-2012-076 | - | KC485128 | KC485161 | KC485192 | KC485219 | KC485251 |
| *P. laurifolia* Ledeb. | Fuyun, Xinjiang,China/1/LiuJQ-XJ-2011-018 | KC485092 | KC485118 | KC485151 | KC485181 | KC485212 | KC485241 |
|  | Burqin, Xinjiang,China/1/LiuJQ-XJ-2011-049 | KC485092 | KC485118 | KC485151 | KC485182 | KC485212 | KC485241 |
|  | Yumin, Xinjiang,China/1/LiuJQ-XJ-2011-082 | KC485094 | KC485119 | KC485152 | KC485183 | KC485212 | KC485242 |
|  | Hami, Xinjiang,China/1/LiuJQ-FJJ-0247-091 | KC485094 | KC485119 | KC485152 | KC485183 | KC485212 | KC485242 |
|  | Beitun, Xinjiang,China/1/LiuJQ-XJ-2011-028 | KC485093 | KC485118 | KC485151 | KC485181 | KC485212 | KC485241 |
| *P. pilosa* Rehd. | Altay, Xinjiang,China/2/LiuJQ-XJ-2011-044-1 | KC485097 | KC485122 | KC485155 | KC485186 | KC485215 | KC485244 |

Continued

Table 1．Continued

| Taxa | Sources/individuals/vouchers | GenBank accession numbers | | | | | |
| --- | --- | --- | --- | --- | --- | --- | --- |
|  |  | *ITS* | *mat*K | *psb*A*-trn*H | *psb*K*-psb*I | *rbc*L | *trnG-psbK* |
|  | Altay, Xinjiang,China/2/LiuJQ-XJ-2011-044-2 | KC485098 | KC485122 | KC485155 | KC485186 | KC485215 | KC485244 |
|  | Yiwu, Xingjiang,China/1/LiuJQ-FJJ-0253-098 | KC485099 | KC485123 | KC485156 | KC485187 | KC485215 | KC485245 |
| *P. talassica* Kom. | Urumqi,Xinjiang,China/1/LiuJQ-XJ-2011-001 | KC485105 | KC485132 | KC485165 | KC485196 | KC485228 | KC485255 |
|  | Urumqi,Xinjiang,China/1/LiuJQ-XJ-2011-002 | KC485106 | KC485132 | KC485165 | KC485196 | KC485228 | KC485255 |
|  | Hejing, Xinjiang,China/1/LiuJQ-XJ-2011-116 | KC485106 | KC485132 | KC485165 | KC485196 | KC485228 | KC485255 |
|  | Hami, Xinjiang,China/1/LiuJQ-FJJ-0251-096 | KC485106 | KC485132 | KC485165 | KC485196 | KC485228 | KC485255 |
|  | Hejing, Xinjiang,China/1/ Liujq-FJJ-0269-122 | KC485107 | KC485132 | KC485165 | KC485197 | KC485228 | KC485255 |
| *P. szechuanica* Schneid. | Xunhua, qinghai, china/1/RY039 | KC485104 | KC485131 | KC485164 | KC485193 | KC485220 | KC485253 |
|  | Minhe, qinghai, china/1/RY047 | KC485104 | KC485130 | KC485164 | KC485195 | KC485221 | KC485254 |
|  | Huzhu, qinghai, china/1/RY062 | KC485104 | KC485130 | KC485164 | KC485195 | KC485221 | KC485254 |
|  | Qilian, qinghai, china/1/RY077 | KC485104 | KC485131 | KC485164 | KC485193 | KC485220 | KC485253 |
|  | Luhuo,Sichuan, China/1/LiuJQ-QTP-2011-260 | - | KC485129 | KC485163 | KC485193 | KC485220 | KC485253 |
|  | Markang, Sichuan,China/1/LiuJQ-QTP-2011-271 | - | KC485130 | KC485164 | KC485194 | KC485221 | KC485254 |
|  | Markang, Sichuan,China/1/LiuJQ-QTP-2011-272 | KC485104 | KC485130 | KC485164 | KC485195 | KC485221 | KC485254 |
| *P. qamdoensis* C. Wang et Tung | Qushui,Xizang,China /1/LiuJQ-QTP-2011-015 | KC485101 | KC485124 | KC485158 | KC485190 | KC485217 | KC485248 |
| *P. ciliata* Wall. | Jilong, Xizang,China/1/LiuJQ-QTP-2011-092 | KC485086 | KC485114 | KC485144 | KC485173 | KC485207 | KC485233 |
| *P. wuana* C. Wang et Tung | Tongmai, Xizang,China/1/LiuJQ-QTP-2011-195 | - | KC485135 | KC485168 | KC485200 | KC485224 | KC485260 |
|  | Bomi, Xizang,China/1/LiuJQ-QTP-2011-206 | KC485109 | KC485135 | KC485168 | KC485200 | KC485224 | KC485260 |
| *P. yatungensis*(C.Wang et P.Y.Fu) C. Wang et Tung | Yadong, Xizang,China/1/LiuJQ-QTP-2011-127 | KC485110 | KC485136 | KC485169 | KC485201 | KC485225 | KC485261 |
|  | Gongbujiangda, Xizang,China/1/LiuJQ-QTP-2011-152 | KC485110 | KC485137 | KC485169 | KC485202 | KC485226 | KC485262 |
|  | Linzhi, Xizang,China/1/LiuJQ-QTP-2011-153 | KC485110 | KC485137 | KC485169 | KC485202 | KC485227 | KC485262 |

Continued

Table 1．Continued

| Taxa | Sources/individuals/vouchers | GenBank accession numbers | | | | | |
| --- | --- | --- | --- | --- | --- | --- | --- |
|  |  | *ITS* | *mat*K | *psb*A*-trn*H | *psb*K*-psb*I | *rbc*L | *trnG-psbK* |
|  | Linzhi, Xizang,China/1/LiuJQ-QTP-2011-160 | - | KC485137 | KC485169 | KC485202 | KC485227 | KC485262 |
|  | Milin, Xizang,China/1/LiuJQ-QTP-2011-162 | KC485110 | KC485137 | KC485169 | KC485202 | KC485227 | KC485262 |
|  | Langxian, Xizang,China/1/LiuJQ-QTP-2011-174 | - | KC485137 | KC485169 | KC485202 | KC485227 | KC485262 |
|  | Linzhi, Xizang,China/1/LiuJQ-QTP-2011-176 | KC485110 | KC485137 | KC485169 | KC485202 | KC485227 | KC485262 |
|  | Bomi, Xizang,China/1/LiuJQ-QTP-2011-192 | KC485110 | KC485137 | KC485169 | KC485202 | KC485227 | KC485262 |
|  | Tongmai, Xizang,China/1/LiuJQ-QTP-2011-197 | KC485110 | KC485137 | KC485169 | KC485202 | KC485227 | KC485262 |
| Sect. *Aigeiros* Duby |  |  |  |  |  |  |  |
| *P. nigra* L. | Fuhai, Xinjiang,China/1/LiuJQ-XJ-2011-031 | KC485095 | KC485120 | KC485153 | KC485184 | KC485213 | KC485243 |
|  | Beitun, Xinjiang,China/1/LiuJQ-XJ-2011-037 | KC485095 | KC485120 | KC485153 | KC485184 | KC485213 | KC485243 |
|  | Altay, Xinjiang,China/1/LiuJQ-XJ-2011-047 | KC485095 | KC485120 | KC485153 | KC485184 | KC485213 | KC485243 |
|  | Burqin, Xinjiang,China/1/LiuJQ-XJ-2011-054 | KC485095 | KC485120 | KC485153 | KC485184 | KC485213 | KC485243 |
|  | 185tuan, Xinjiang,China/1/LiuJQ-XJ-2011-070 | KC485095 | KC485120 | KC485153 | KC485184 | KC485213 | KC485243 |
| *P. × jrtyschensis* Ch. Y. Yang | Fuyun, Xinjiang,China/1/LiuJQ-XJ-2011-025 | KC485091 | KC485116 | KC485149 | KC485177 | KC485210 | KC485239 |
|  | Altay, Xinjiang,China/2/LiuJQ-XJ-2011-046-1 | KC485091 | KC485117 | KC485150 | KC485178 | KC485211 | KC485240 |
|  | Altay, Xinjiang,China/2/LiuJQ-XJ-2011-046-2 | KC485091 | KC485117 | KC485149 | KC485179 | KC485210 | KC485239 |
|  | 185tuan, Xinjiang,China/1/LiuJQ-XJ-2011-067 | KC485091 | KC485116 | KC485149 | KC485177 | KC485210 | KC485239 |
|  | Habahe, Xinjiang,China/1/LiuJQ-XJ-2011-073 | KC485091 | KC485116 | KC485149 | KC485177 | KC485210 | KC485239 |
|  | Beitun, Xinjiang,China/1/LiuJQ-XJ-2011-030 | - | KC485116 | KC485149 | KC485180 | KC485210 | KC485239 |
| *P. afghanica* (Aitch.et Hemsl.) Schneid. | Uqia, Xinjiang,China/2/LiuJQ-FJJ-0209-010-1,-2 | KC485082 | KC485111 | KC485140 | KC485170 | KC485203 | KC485229 |
|  | Pishan, Xinjiang,China/1/LiuJQ-FJJ-0227-048 | KC485082 | KC485111 | KC485140 | KC485170 | KC485203 | KC485229 |
| *P. pamirica* var. *akqiensis* C.Y.Yang | Akqi, Xinjiang,China/1/LiuJQ-FJJ-0204-005 | KC485096 | KC485121 | KC485154 | KC485185 | KC485214 | KC485256 |

Continued

Table 1．Continued

| Taxa | Sources/individuals/vouchers | GenBank accession numbers | | | | | |
| --- | --- | --- | --- | --- | --- | --- | --- |
|  |  | *ITS* | *mat*K | *psb*A*-trn*H | *psb*K*-psb*I | *rbc*L | *trnG-psbK* |
| Sect. *Turanga* Bge. |  |  |  |  |  |  |  |
| *P. euphratica* Oliv. | Ejinaqi, Neimeng,China/1/ EJNQ 25 | KC485089 | KC485138 | KC485147 | KC485175 | KC485209 | KC485236 |
|  | Minqin, Gansu,China/1/MQ59 | KC485089 | KC485138 | KC485148 | KC485176 | KC485209 | KC485237 |
|  | Shawan, Xinjiang,China/1/SHW69 | KC485088 | KC485138 | KC485148 | KC485176 | KC485209 | KC485237 |
|  | Moyu, Xinjiang,China/1/LiuJQ-XJ-2011-099 | KC485090 | KC485138 | KC485147 | KC485175 | KC485209 | KC485238 |
|  | Lunnan, Xinjiang,China/1/LiuJQ-XJ-2011-117 | KC485088 | KC485138 | KC485147 | KC485175 | KC485209 | KC485236 |
| *P. pruinosa* Carr. | Awati, Xinjiang,China/1/LiuJQ-XJ-2011-090 | KC485100 | KC485139 | KC485157 | KC485188 | KC485216 | KC485246 |
|  | Maigaiti, Xinjiang,China/1/LiuJQ-XJ-2011-097 | KC485100 | KC485139 | KC485157 | KC485188 | KC485216 | KC485246 |
|  | Moyu, Xinjiang,China/1/LiuJQ-XJ-2011-100 | KC485100 | KC485139 | KC485157 | KC485188 | KC485216 | KC485246 |
|  | Minfeng, Xinjiang,China/1/LiuJQ-XJ-2011-103 | KC485100 | KC485139 | KC485157 | KC485189 | KC485216 | KC485247 |
|  | Lunnan, Xinjiang,China/1/LiuJQ-XJ-2011-118 | KC485100 | KC485139 | KC485157 | KC485189 | KC485216 | KC485247 |
|  | Shaya, Xinjiang,China/1/LiuJQ-XJ-2011-119 | KC485100 | KC485139 | KC485157 | KC485188 | KC485216 | KC485246 |

“-”means the sequencing is failed.
