# Supplementary material for: Barcoding Poplars (Populus L.) from Western China
Source: PLoS One. 2013 Aug 19;8(8):e71710. doi: 10.1371/journal.pone.0071710 (PMC3747233; doi:10.1371/journal.pone.0071710)
Supplement: Table S2 — Primers used for amplification and sequencing. (DOCX) [file pone.0071710.s003.docx]

**Table S2**. Primers used for amplification and sequencing

| Regions | Successful primers and their sequences (5’-3’) | | References |
| --- | --- | --- | --- |
| *mat*K | 3F_KIM | TAATGAGAAAGATTTCTGCATATACG | Schroeder et al. 2012 [1] |
|  | 1R_KIM | TTTACGATCAATTCATTCAATATTTCC | Schroeder et al. 2012 [1] |
| *psb*A*-trn*H | trnH2 | CGCGCATGGTGGATTCACAATCC | Tate & Simpson 2003 [2] |
|  | psbAF | GTTATGCATGAACGTAATGCTC | Sang et al. 1997 [3] |
| *rbc*L | F | ATGTCACCACAAACAGAGACT | This study |
|  | R | CCGAATTGTAGTACGGAATC | This study |
| *trn*G-*psb*K | 3a | GAAGGATTCGAACCTCCGAATG | Schroeder et al. 2011 [1] |
|  | 3b | CTGGCATAACATCTACGATTGG | Schroeder et al. 2011 [1] |
| *psb*K-*psb*I | 4a | CCAATCGTAGATGTTATGCCAG | Schroeder et al. 2011 [1] |
|  | 4b | GGATTACGCCCTGGATCATTAG | Schroeder et al. 2011 [1] |
| ITS | PopF1a | ACCTGCCTAGCAGAACGAC | This study |
|  | PopF2a | AAGGATCATTGTCGARACCTG | This study |
|  | ITS 4 | TCCTCCGCTTATTGATATGC | White et al. 1990 [4] |

**References:**

1. Schroeder H, Hoeltken AM, Fladung M (2012) Differentiation of Populus species using chloroplast single nucleotide polymorphism (SNP) markers–essential for comprehensible and reliable poplar breeding. Plant Biol (Stuttg) 14: 374-381.

2. Tate JA, Simpson BB (2003) Paraphyly of *Tarasa* (Malvaceae) and diverse origins of the polyploidy species. Syst Bot 28: 723-737.

3. Sang T, Crawford DJ, Stuessy TF (1997) Chloroplast DNA phylogeny, reticulate evolution, and biogeography of *Paeonia* (Paeoniaceae). Am J Bot 84: 1120-1136.

4. White TJ, Burns T, Lee S, Taylor J (1990) Amplification and direct sequencing of fungal ribosomal RNA genes for phylogenetics. In: Innis M, Gelfand D, Sninsky J, White T, editors. PCR protocols: a guide to methods and applications. San Diego: Academic Press. pp. 315-322.
